# Candida Femoral Osteomyelitis in a Young Adult Without Known Risk Factors for Immunosuppression: A Case Report

**DOI:** 10.7759/cureus.107308

**Published:** 2026-04-18

**Authors:** Ahmed M Albushtra, Abdulsalam Mohsen, Abdulwahed Abdulkareem, Younes Aljobahi, Abdullah Obaid, Ashraf Al-ferzai

**Affiliations:** 1 Department of Orthopedic Surgery, School of Medicine, Ibb University, Ibb, YEM; 2 Department of Orthopedic Surgery, School of Medicine, Aden University, Aden, YEM

**Keywords:** candida, case report, chronic osteomyelitis, femur, fungal osteomyelitis, immunocompetent

## Abstract

Fungal osteomyelitis is a rare and challenging infection that typically affects individuals with compromised immune systems. Its insidious presentation often leads to delays in diagnosis and treatment. We report *Candida *species femoral osteomyelitis in a previously healthy 20-year-old man without known risk factors for immunosuppression who presented with a six-month history of unilateral thigh pain and limping. Laboratory tests, including C-reactive protein (mildly elevated at 10 mg/L) and erythrocyte sedimentation rate (20 mm/hour), were near‑normal. Magnetic resonance imaging (MRI) revealed a lytic lesion in the femoral diaphysis with associated cortical destruction and periosteal reaction. The patient underwent surgical debridement and bone biopsy. Intraoperative cultures grew *Candida* species, confirmed by histopathology demonstrating inflamed fibrovascular tissue without evidence of malignancy. Treatment consisted of oral ketoconazole 400 mg daily for six months, resulting in complete clinical recovery with radiographic evidence of bone healing at one‑year follow-up. In conclusion, this case underscores the critical need to consider fungal etiologies in the differential diagnosis of subacute osteomyelitis, even in young patients without traditional risk factors. A high index of suspicion, combined with early surgical intervention and targeted antifungal therapy, is paramount for achieving a favorable clinical outcome and preventing long‑term morbidity.

## Introduction

Osteomyelitis is a complex inflammation of bone, mainly caused by bacteria. Fungal osteomyelitis, however, is extremely rare, accounting for less than 1% of all reported bone infections [1]. These infections typically affect patients with weakened immune systems, such as those with poorly controlled diabetes, HIV, hematologic malignancies, or neutropenia, or those receiving long-term steroid or immunosuppressive therapy. Intravenous drug use and the presence of vascular catheters are additional risk factors for infection spread [2,3].

Among fungal pathogens, *Candida* species are most frequently identified in fungal osteomyelitis [4]. The clinical importance of *Candida* speciation is considerable, as antifungal resistance profiles vary significantly between species (e.g., *C. glabrata* and *C. krusei* demonstrate reduced susceptibility to fluconazole compared to *C. albicans*) [2]. Infection usually occurs following hematogenous spread or direct inoculation during trauma or surgery. The course of fungal osteomyelitis is often slow and subacute, resulting in mild systemic symptoms and general inflammatory markers. This leads to low clinical awareness, causing delays in diagnosis that may result in permanent bone damage and loss of function [3,5].

While much of the existing research focuses on immunocompromised patients, the presentation of fungal bone infections in healthy individuals remains poorly understood. Only a few case reports worldwide describe such occurrences. These unusual cases complicate standard diagnostic procedures and highlight the need for early invasive measures, such as biopsy and culture, to distinguish this type of infection from chronic bacterial osteomyelitis or primary bone malignancies [6,7].

In this report, we describe a rare case of isolated *Candida* femoral osteomyelitis in a healthy 20-year-old man who had no known risk factors for immune suppression.

## Case presentation

A 20-year-old healthy man presented with a six-month history of progressive left thigh pain and associated limp, worsened by activity, along with night chills and low-grade fever. He reported intermittent use of non-steroidal anti-inflammatory drugs and denied any significant medical history, recent or prolonged antibiotic use, corticosteroid therapy, intravenous drug use, or traditional risk factors for immunosuppression. While he reported exposure to pigeon droppings, this was considered in the differential diagnosis for *Cryptococcus neoformans* rather than as a primary risk factor for *Candida* infection.

**Figure 1:**
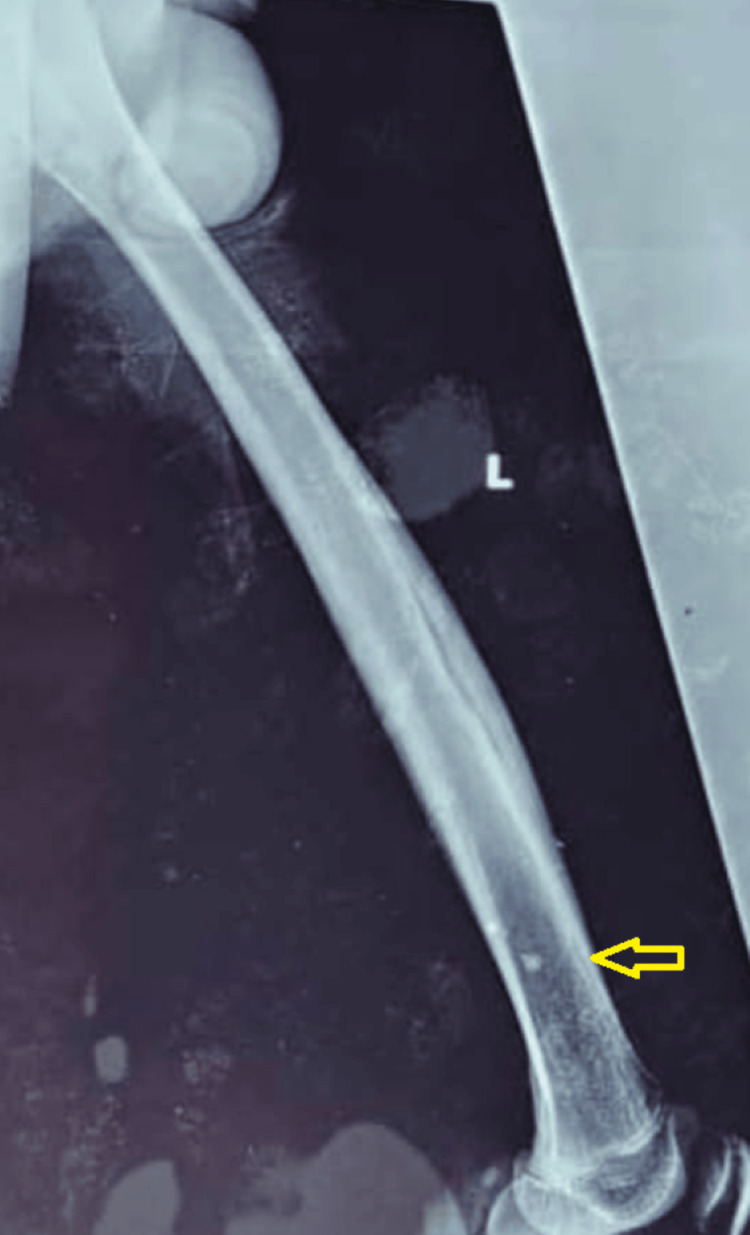
Plain radiography of the left femur (anteroposterior view) The radiograph demonstrates marked cortical thickening and sclerosis localized to the mid-to-distal diaphysis (yellow arrows). An elongated, radiolucent intracortical track is visible, representing a cloaca or chronic tract, alongside irregular medullary density. These features are characteristic of subacute to chronic osteomyelitis, with no evidence of acute pathological fracture or knee joint involvement.

Physical examination revealed a stable patient with low-grade fever (37.9°C) and marked tenderness and warmth in the left thigh, while hip and knee joint motion remained intact. Initial laboratory tests were largely normal, with a white blood cell count of 8,000/μL, random blood glucose of 95 mg/dL, and an erythrocyte sedimentation rate within limits at 20 mm/hour. C-reactive protein was mildly elevated at 10 mg/L. Serologies for HIV, hepatitis B, and hepatitis C were negative. Blood cultures were not obtained due to limited diagnostic availability. Plain radiographs of the left femur revealed a suspicious lytic lesion measuring approximately 3.5 × 2 cm in the mid-diaphysis (Figure 1).

**Figure 2:**
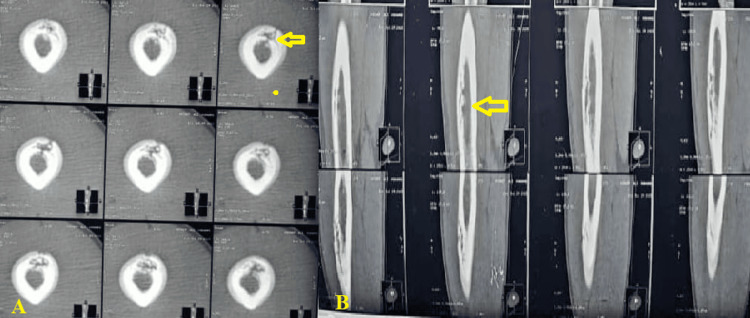
CT of the left femur Coronal reformatted image (A) and axial slice (B) at the level of the mid-diaphysis. The images reveal a well-defined sequestrum (a fragment of dense, necrotic bone) located within a prominent cloaca or drainage tract (yellow arrows). There is evidence of extensive circumferential periosteal reaction and involucrum formation (a layer of new bone growth surrounding the infected area), resulting in significant cortical thickening and narrowing of the medullary canal. These combined findings are characteristic of chronic osteomyelitis. CT: computed tomography

Computed tomography (CT) confirmed a mixed lytic and sclerotic lesion with focal cortical thickening, a prominent periosteal reaction, and altered medullary density (Figure 2).

**Figure 3:**
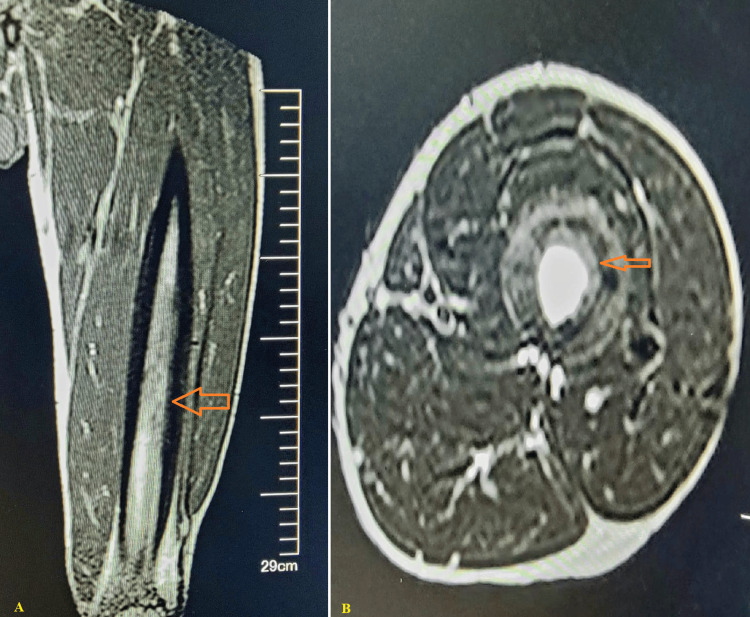
MRI of the left femur Sagittal T1-weighted (A) and axial T2-weighted (B) sequences. (A) An area of hypointense marrow signal (darkened area) within the medullary canal (orange arrow). (B) A corresponding focus of marked hyperintense signal (bright white area) (orange arrow), consistent with fluid collection or pus within an intraosseous abscess (Brodie’s abscess). The surrounding hyperintensity in the vastus musculature represents moderate reactive soft tissue edema, a common inflammatory response to underlying bone infection. MRI: magnetic resonance imaging

Magnetic resonance imaging (MRI) further characterized the lesion, demonstrating cortical destruction and periosteal involvement, with the lesion appearing hypointense on T1-weighted sequences and hyperintense on T2-weighted sequences with post-contrast enhancement (Figure 3).

Given the atypical presentation and imaging findings mimicking primary bone malignancy, the patient underwent surgical debridement and biopsy for definitive diagnosis.

Intraoperatively, purulent material was encountered. Microscopic examination of the specimen revealed abundant pus cells, abundant red blood cells, and Gram-positive budding yeast forms. Cultures were performed on Sabouraud dextrose agar and confirmed the growth of *Candida* species; however, specific speciation (e.g., *C. albicans* versus non-albicans species) was not performed due to limitations in our institutional laboratory facilities.

Antifungal susceptibility testing was performed using the disk diffusion method on Mueller‑Hinton agar supplemented with glucose and methylene blue per CLSI M44‑A2 guidelines. The isolate demonstrated sensitivity to ketoconazole, miconazole, and clotrimazole (all 3+), and a lower degree of sensitivity to fluconazole (1+), while exhibiting resistance to voriconazole. Histopathological analysis of bone samples showed cortical-type thick bone trabeculae with fibrovascular tissue in between, and no evidence of malignancy. A diagnosis of isolated fungal osteomyelitis of the femur due to *Candida* species was established.

Postoperatively, the patient received a six-month course of oral ketoconazole (400 mg daily). This therapeutic choice was based on the strong in vitro susceptibility (3+) to ketoconazole, local antifungal availability, and resource considerations, despite this agent no longer being considered first-line therapy per current Infectious Diseases Society of America (IDSA) guidelines. Liver function tests were monitored monthly during ketoconazole therapy and remained within normal limits throughout treatment. A five-day course of empirical intravenous ceftriaxone (2 g daily) was administered.

**Figure 4:**
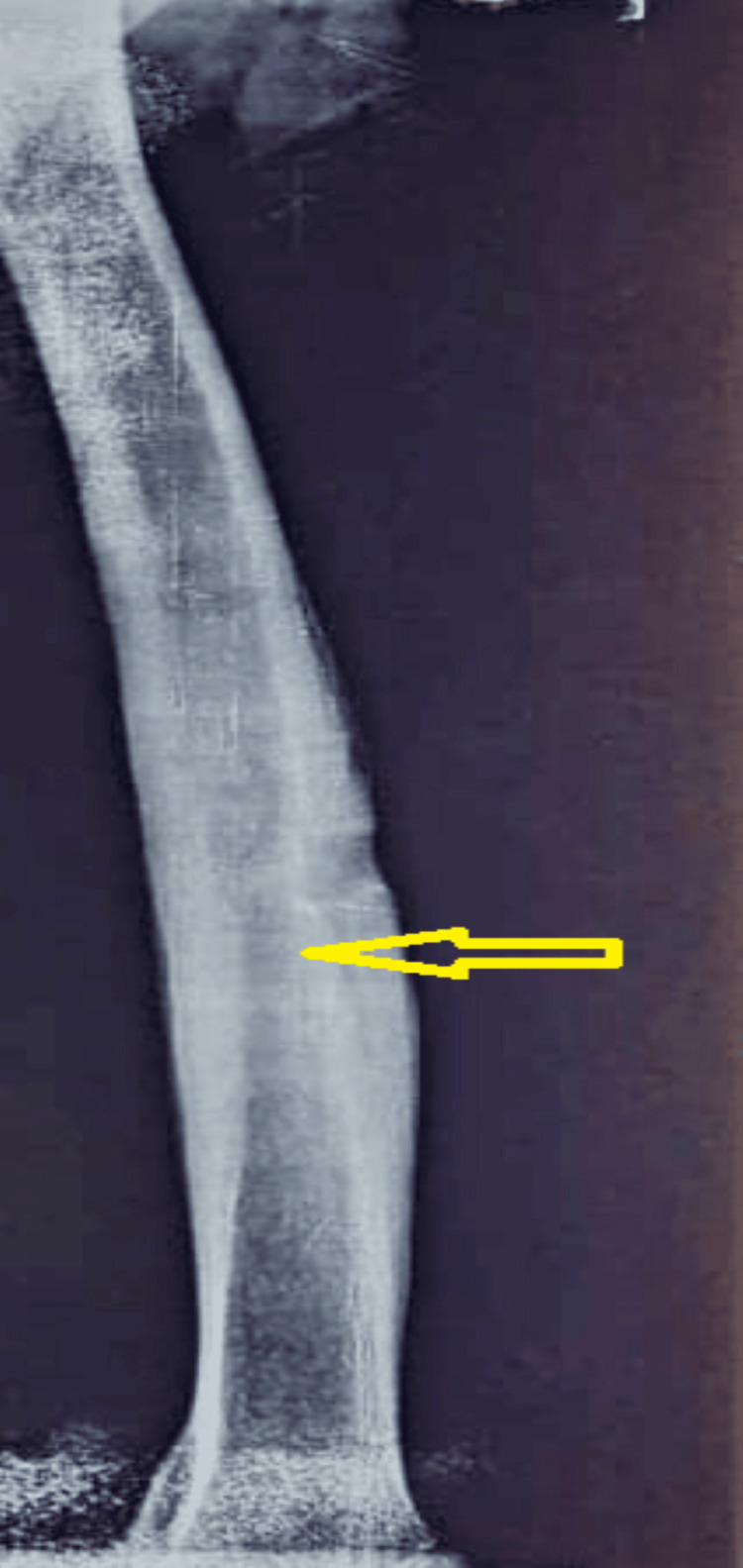
Follow-up radiograph of the left femur obtained one year post-treatment The anteroposterior view demonstrates significant cortical remodeling and progressive ossification of the previously noted lytic regions. There is a marked reduction in periosteal thickening and normalization of the medullary density compared to the initial presentation. No new lytic lesions, sequestra, or radiographic signs of fungal recurrence are identified (yellow arrow), confirming a successful long-term response to combined surgical debridement and antifungal therapy.

Over six months of follow-up, the patient demonstrated significant clinical improvement with complete symptom resolution (pain score reduced to 0/10) and normalization of inflammatory markers (erythrocyte sedimentation rate < 10 mm/hour at six months). Follow-up radiographs at three and six months showed progressive bone healing, and one-year follow-up imaging confirmed continued resolution without clinical or radiographic evidence of recurrence (Figure 4). The patient continues surveillance with the orthopedic team at three-month intervals to monitor for late recurrence.

## Discussion

Fungal osteomyelitis represents a rare but clinically significant entity, accounting for less than 1% of all bone infections [8]. The epidemiological landscape of mycotic bone infections has been significantly influenced by the global HIV epidemic, which has been associated with increased incidence of fungal-related musculoskeletal pathologies. Currently, fungal pathogens are implicated in approximately 1% of all osteomyelitis cases, increasing to 4% in infectious arthritis [3]. Despite this, the literature remains dominated by isolated case reports and limited case series, particularly regarding *Candida*-specific bone involvement [4].

A comprehensive analysis of 112 documented cases of *Candida* musculoskeletal infections identified a median patient age of 40 years, with a male predominance (62%). While many affected individuals were immunocompetent, the majority possessed distinct risk factors, including prior surgery (35%), hematological malignancies (16%), solid organ transplantation (9%), trauma (9%), and intravenous drug use (9%) [9]. A separate review of 207 evaluable cases of *Candida* osteomyelitis reported a median age of 30 years, with a male-to-female ratio exceeding 2:1. Notably, 90% of these patients were not neutropenic, suggesting that infection frequently occurs in non‑severely immunocompromised individuals [4]. While vertebral involvement is common in adults, the femur is recognized as a primary site of infection in pediatric and younger populations [10], a finding consistent with our patient’s presentation.

In contrast, our patient was a 20‑year‑old man with no reported history of medical illness, trauma, or surgical intervention. The absence of classic risk factors, including diabetes mellitus, HIV, immunosuppressive therapy, or intravenous drug use, makes this case unusual. Similar reports of *Candida* osteomyelitis in immunocompetent individuals without predisposing factors have been described [11]. Oksi et al. reported a case of *Candida *dubliniensis spondylodiscitis in an otherwise healthy adult, confirming that even rare *Candida* species can cause bone infection in the absence of immune defects [12]. Mardi and Sharma described an 11‑year‑old immunocompetent boy with right knee septic arthritis and multifocal distal femoral osteomyelitis [5]. However, the possibility of indolent or unrecalled risk factors such as transient fungemia from a minor gastrointestinal or mucosal breach, or unrecognized mild immunodeficiency, cannot be entirely excluded [4,10]. Furthermore, the omission of a comprehensive immunologic workup, such as immunoglobulin levels, represents a limitation of this study.

*Candida* species are well-recognized commensals inhabiting the human gastrointestinal tract, genital tract, and cutaneous surfaces [13]. While various species, including *C. albicans*, *C. glabrata*, and *C. tropicalis*, are known to cause bone infections, they typically require a predisposing factor, such as indwelling intravenous catheters, broad-spectrum antibiotic use, or underlying immunodeficiency [3]. However, mirroring the present observation, recent studies indicate that a significant subset of patients present with no apparent immune deficiency [9]. This was consistent with our patient, whose viral markers and renal function were entirely normal, yet he developed primary fungal osteomyelitis of the femur.

The primary pathogenic route for fungal osteomyelitis is hematogenous dissemination. The highly vascularized nature of the medullary canal provides a gateway for blood-borne fungi to colonize bone tissue. The mammalian synovium is extremely vascular and lacks a basement membrane, facilitating direct microbial access to the synovial space and permitting hematogenous inoculation of even healthy joints [4]. In our case, blood cultures were not obtained due to limited diagnostic availability, which precluded the direct assessment of potential hematogenous dissemination. Alternative mechanisms include direct inoculation following trauma or surgical procedures, as well as contiguous spread from adjacent infectious foci [4,5].

Diagnosing fungal osteomyelitis is often complicated by a significant latency period; symptoms may emerge weeks to months after initial seeding [5,14]. Our patient presented with a six-month history of dull pain and limp, a subacute progression characteristic of fungal pathogens. Clinical manifestations frequently mimic bone tumors, as evidenced by the radiological mimicry of malignancy in this case. Radiological examination findings are often inconclusive, as mixed lytic-sclerotic lesions and periosteal reactions lack specificity and closely mimic both chronic bacterial osteomyelitis and primary bone malignancies [13].

Synovial fluid analysis typically reveals an inflammatory exudate with neutrophilic predominance, indistinguishable from pyogenic arthritis. Direct Gram staining visualizes fungal elements in only 20% of cases, whereas culture of tissue specimens yields significantly higher diagnostic sensitivity. Blood cultures are frequently negative [4,5]. In our case, the diagnosis was confirmed by culturing purulent material from the surgical biopsy, which grew *Candida* spp. However, species‑level identification was not feasible due to limited laboratory resources. Although conventional methods like CHROMagar are cost‑effective, they have a misidentification rate of approximately 14% and often fail to distinguish non‑albicans species [15]. This distinction is crucial, as antifungal susceptibility varies by species. For instance, *C. glabrata* and *C. krusei* are frequently resistant to fluconazole [10].

In our case, antifungal susceptibility testing by disk diffusion showed sensitivity to ketoconazole but resistance to voriconazole. This pattern is atypical for *Candida* spp., which are usually susceptible to voriconazole despite fluconazole cross‑resistance in some non‑albicans strains [16]. This discrepancy likely reflects a testing artifact rather than true resistance. Lacking advanced confirmatory methods, we opted for ketoconazole, an effective and affordable choice in resource‑limited settings.

Histopathological examination of bone fragments revealed thick cortical bone trabeculae with intervening fibrovascular tissue and no evidence of malignancy. While granulomas are rarely present in *Candida* infection [2,3,6], our biopsy confirmed the absence of neoplastic cells, effectively ruling out primary bone tumors.

The therapeutic objectives in fungal osteomyelitis encompass pathogen eradication, symptom relief, prevention of articular destruction, and functional restoration. Due to the scarcity of fungal bone infections, evidence‑based treatment standards are still evolving [3,4]. Current Infectious Diseases Society of America guidelines recommend fluconazole (400 mg daily) for a minimum of six weeks, or lipid formulation amphotericin B (5 mg/kg daily) for at least two weeks, followed by fluconazole to complete therapy [17].

Treatment durations frequently extend beyond six weeks. Voriconazole and posaconazole demonstrate comparable efficacy to fluconazole against *C. albicans* while offering enhanced activity against fluconazole‑resistant species such as *C. glabrata* and *C. krusei* [2].

In our case, the isolate demonstrated strong sensitivity to ketoconazole, which directed our pharmacological strategy. Although ketoconazole has largely been superseded by newer triazoles due to hepatotoxicity concerns, it remains effective in resource‑limited settings where alternative agents may be unavailable. This was consistent with a prior report by Bannatyne and Clarke, who successfully treated a three‑year‑old immunocompetent child with post‑traumatic *Candida albicans* osteomyelitis using a three‑month course of oral ketoconazole [18]. With monthly liver function monitoring, ketoconazole succeeded here as well.

Antibiotics were used judiciously to prevent secondary bacterial infection, which complicates approximately 12% of *Candida* osteomyelitis cases [10]. A five‑day course of ceftriaxone covered this risk and was discontinued after confirming pure fungal growth. This step is critical, as unnecessary antibiotics can promote fungal overgrowth by disrupting competing bacterial flora. Empirical ceftriaxone was administered to cover potential bacterial superinfection, a decision supported by the documented 12% rate of concomitant bacterial co‑infection in *Candida* osteomyelitis [4].

The role of surgical intervention is critical in fungal osteomyelitis. Intraoperatively, purulent material suggested biofilm formation, a resilient microbial community that promotes drug resistance and immune evasion. Thus, thorough surgical debridement proved as vital as antifungal therapy for cure [4,10]. Gamaletsou et al. reported that combined surgery and antifungal therapy were employed in 48% of *Candida* osteomyelitis cases, with an overall complete response rate of 32%, reflecting the difficulty in treating this infection [4]. Relapsed infection, possibly related to inadequate treatment duration, occurred in 32% of patients who ultimately achieved complete response [4]. This high rate of late recurrence justifies the continued clinical and radiographic surveillance of our patient at regular intervals. Slenker et al., in their review of 211 cases of *Candida* osteomyelitis, reported an overall treatment success rate of 91%, with 75% of patients achieving cure within six months [7]. While some data suggest medical treatment alone may be attempted, the risk of dissemination and relapse makes a compelling case for combined surgical and medical management [2,4].

In our patient, the combination of thorough surgical debridement and a six‑month course of oral ketoconazole resulted in excellent clinical and biological outcomes, evidenced by complete symptom resolution, normalization of inflammatory markers, and confirmatory imaging at follow‑up. This success illustrates the efficacy of a dual‑modality approach in healthy young adults and highlights the necessity of early MRI and culture‑based diagnosis.

## Conclusions

Fungal osteomyelitis of the femur caused by *Candida* species in a healthy young adult is an extremely rare event that presents significant diagnostic challenges. In this case, the subacute progression and inconclusive radiologic findings closely mimicked primary bone malignancy, necessitating early surgical intervention and tissue biopsy for definitive diagnosis. This report illustrates that unusual fungal pathogens should be considered in the differential diagnosis of atypical osteomyelitis, even in patients without traditional risk factors. Ultimately, a combined approach of thorough surgical debridement and susceptibility-guided antifungal therapy proved fundamental to achieving complete clinical resolution.
